# Experimental evidence for opposing effects of high deer density on tick-borne pathogen prevalence and hazard

**DOI:** 10.1186/s13071-021-05000-0

**Published:** 2021-09-30

**Authors:** Sara Gandy, Elizabeth Kilbride, Roman Biek, Caroline Millins, Lucy Gilbert

**Affiliations:** 1 Institute of Biodiversity, Animal Health and Comparative Medicine, University of Glasgow, Glasgow, UK; 2 The James Hutton Institute, Craigiebuckler, Aberdeen, UK; 3 Institute of Infection, Veterinary and Ecological Sciences, University of Liverpool, Liverpool, UK

**Keywords:** *Borrelia burgdorferi* sensu lato, Dilution effect, Lyme disease, Ecological cascades, *Ixodes ricinus*

## Abstract

**Background:**

Identifying the mechanisms driving disease risk is challenging for multi-host pathogens, such as *Borrelia burgdorferi* sensu lato (s.l.), the tick-borne bacteria causing Lyme disease. Deer are tick reproduction hosts but do not transmit *B. burgdorferi* s.l., whereas rodents and birds are competent transmission hosts. Here, we use a long-term deer exclosure experiment to test three mechanisms for how high deer density might shape *B. burgdorferi* s.l. prevalence in ticks: increased prevalence due to higher larval tick densities facilitating high transmission on rodents (M1); alternatively, reduced *B. burgdorferi* s.l. prevalence because more larval ticks feed on deer rather than transmission-competent rodents (dilution effect) (M2), potentially due to ecological cascades, whereby higher deer grazing pressure shortens vegetation which decreases rodent abundance thus reducing transmission (M3).

**Methods:**

In a large enclosure where red deer stags were kept at high density (35.5 deer km^−2^), we used an experimental design consisting of eight plots of 0.23 ha, four of which were fenced to simulate the absence of deer and four that were accessible to deer. In each plot we measured the density of questing nymphs and nymphal infection prevalence in spring, summer and autumn, and quantified vegetation height and density, and small mammal abundance.

**Results:**

Prevalence tended to be lower, though not conclusively so, in high deer density plots compared to exclosures (predicted prevalence of 1.0% vs 2.2%), suggesting that the dilution and cascade mechanisms might outweigh the increased opportunities for transmission mechanism. Presence of deer at high density led to shorter vegetation and fewer rodents, consistent with an ecological cascade. However, Lyme disease hazard (density of infected *I. ricinus* nymphs) was five times higher in high deer density plots due to tick density being 18 times higher.

**Conclusions:**

High densities of tick reproduction hosts such as deer can drive up vector-borne disease hazard, despite the potential to simultaneously reduce pathogen prevalence. This has implications for environmental pathogen management and for deer management, although the impact of intermediate deer densities now needs testing.

**Graphical abstract:**

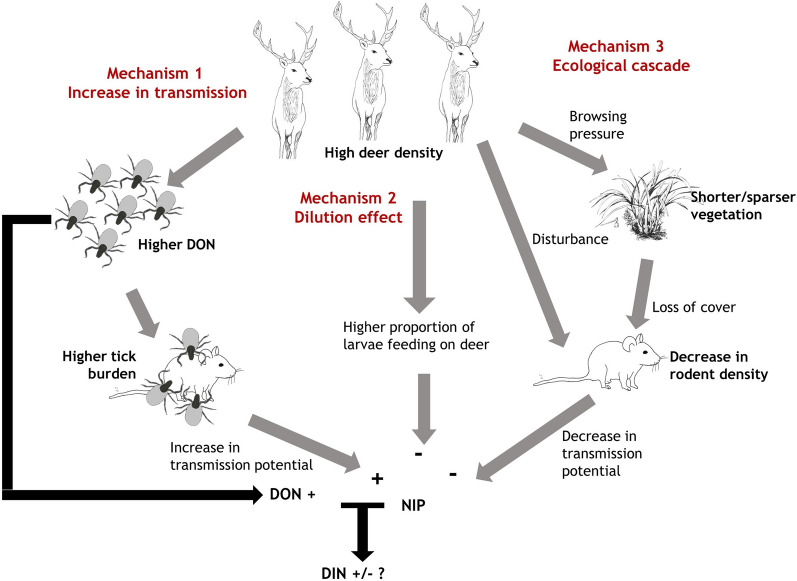

**Supplementary Information:**

The online version contains supplementary material available at 10.1186/s13071-021-05000-0.

## Background

Ecological cascades occur when a change in population or activity of a keystone species precipitates a cascade of alterations in an ecosystem [1]. While there are many examples of ecological cascades, tests of their extension to infectious disease risk have received less attention. This concept could be particularly significant for vector-borne diseases, which rely upon vectors to carry and transmit pathogens from one host to another, and cascading effects could manifest from changes in both vector host and pathogen host populations. For instance, one study showed that the introduction of Burmese pythons (*Python bivittatus*) resulted in a decline in some mammal species but not in those acting as transmission hosts for the mosquito-borne Everglades virus. This increased the proportion of mosquito blood meals taken from transmission hosts [2]. Other researchers found a positive association between coyote (*Canis latrans*) density and Lyme disease incidence because coyotes suppress red fox (*Vulpes vulpes*) populations, causing a release of fox predation on small mammals which increased densities of small mammals that transmit Lyme disease pathogens [3]. Lastly, two studies conducted in the Netherlands investigated the impacts of cattle grazing on small mammals, tick density and tick-borne pathogen (*Borrelia burgdorferi*) prevalence [4, 5]. Gassner et al. [4] observed fewer rodents and ticks in grazed plots while Sprong et al. [5] found no effects of cattle grazing on the density of infected nymphs (DIN).

While these studies demonstrate that trophic interactions can shape vector-borne disease risk, field experimental approaches to dissect these effects remain rare. These include those on wild herbivores, which have been shown to alter habitat [4, 6], with cascading effects on communities of small mammals [7, 8], which are transmission hosts for many pathogens, including tick-borne pathogens (e.g. tick-borne encephalitis virus and the bacteria causing Lyme disease). Here, therefore, we test this potential mechanism alongside other potential mechanisms of how large herbivores might shape tick-borne pathogen prevalence and hazard.

Lyme disease, the most prevalent vector-borne disease in humans in the northern hemisphere, is an emerging disease in Europe and North America [9]. It is caused by the *Borrelia burgdorferi* sensu lato (s.l.) complex of bacteria (hereinafter referred to as *B. burgdorferi* s.l.) and transmitted by ixodid ticks. In Europe, the primary vector is *Ixodes ricinus* [9], a generalist three-host tick that feeds on a wide range of vertebrate species [10]. There are several genospecies of the bacteria within the *B. burgdorferi* s.l. complex, each with different transmission host associations. In northern Europe, rodents transmit *Borrelia afzelii* [11] while birds are associated with *Borrelia valaisiana* and *Borrelia garinii* [12]. *Borrelia burgdorferi* sensu stricto, the fourth most abundant genospecies, can be transmitted by both mammals and birds. Transmission is therefore predicted to increase as more immature ticks feed on infected hosts such as rodents [13, 14].

Deer, on the other hand, do not transmit *B. burgdorferi* s.l. [15, 16]. However, deer are the most important hosts for feeding adult female *I. ricinus* ticks prior to egg laying and, as such, high deer densities are often associated with high tick population densities [16–20]. We can therefore predict that high deer densities may increase tick burdens on transmission hosts such as rodents, thereby increasing pathogen transmission [13]. Deer can also feed immature tick life stages [10], so we could predict that, at high deer densities, a higher proportion of immature ticks feed on deer instead of transmission hosts, thus lowering *B. burgdorferi* s.l. prevalence in the tick population through a dilution effect [21, 22]. An alternative mechanism could also result in lower nymphal infection prevalence (NIP) from high deer densities since deer grazing can lead to sparser vegetation cover [6], with cascading negative effects on the density of transmission hosts such as rodents [7, 8] and birds [23]. While some studies have demonstrated an association between deer abundance and Lyme disease hazard [24, 25], the mechanisms driving this association are equivocal. A particular gap in knowledge that this study aims to fill is the potential role of deer in regulating NIP and Lyme disease hazard through their cascading effects on vegetation and therefore transmission hosts such as rodents. Such cascading effects on vegetation and rodents might be predicted to occur even at small spatial scales as plant communities, herbivore grazing pressure, rodent activity and tick distribution are all spatially highly heterogeneous [24, 26–28]. Consequently, rodents are expected to spend more time in patches with favourable vegetation structure and less grazing pressure. These heterogeneities provide the opportunity to test cascading effects experimentally, at small spatial scales most amenable to manipulation. Here, we use a replicated deer exclosure experiment to test the following three possible mechanisms (M1, M2 and M3) for the effects of high deer density on NIP:

- M1-Increase in transmission potential: We predict that high deer density will lead to a high density of questing larvae which, assuming there are enough hosts in the system to provide blood meals for the larvae, will translate into high nymph densities (density of questing nymphs; DON). For a given transmission host density, this could result in higher larval and nymphal tick burdens on transmission hosts [13], which could result in higher NIP than in areas without deer.
- M2-Dilution effect: We predict lower prevalence in areas of high deer density due to a higher proportion of larval ticks feeding on deer instead of on transmission hosts such as rodents.
- M3-Ecological cascade: We predict that intense grazing pressure due to high deer densities will result in shorter vegetation and, therefore, fewer rodents [6–8] and lower prevalence. Rodent activity might also be negatively affected by high deer density directly through disturbance.

**Fig. 1:**
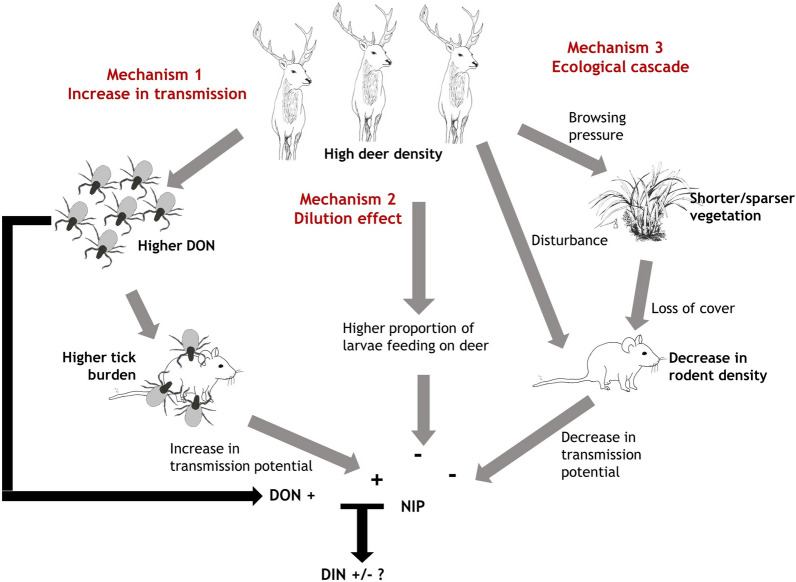
Conceptual diagram to illustrate three pathways through which high deer density might affect nymphal infection prevalence (*NIP*) with *Borrelia burgdorferi* sensu lato (s.l.), and how the density of infected nymphs (*DIN*-Lyme disease hazard) depends on a combination of NIP and the density of questing nymphs (*DON*)

These different mechanisms are not mutually exclusive and the effect of high deer density on NIP will depend on the relative strength of the first (transmission potential) mechanism compared to the other two (dilution and ecological cascade mechanisms) (Fig. 1). A further key aim was to test the impact of high deer density on Lyme disease hazard, which is defined as the DIN in the environment and is the product of NIP and DON [29]. It is difficult to predict the effect of deer on Lyme disease hazard because it will depend on the relative strengths of the effect of deer on DON and the mechanisms driving NIP (Fig. 1).

The use of experimental deer exclosures is particularly suitable for testing the ecological cascades mechanism as it maximises habitat impacts while avoiding the introduction of noise from heterogeneities in land use, habitat, topography and climate that typify landscape-scale surveys. In addition, the exclusion of deer has high applied relevance since deer fencing is a common land management tool and is increasingly being used to mitigate the impacts of ticks [17].

## Methods

### Experimental design

The experiment took place at Glensaugh Research Farm in Aberdeenshire, Scotland (56.914217 N, − 5.532070 E), in a 15-ha enclosure of upland moorland where five red deer (*Cervus elaphus*) stags were kept (density 35.5 deer km^−2^). Deer were supplementary fed during the winter and treated orally against intestinal worms with a broad-spectrum anti-parasite agent (ivermectin) once a year in the autumn. While the effect of this anti-parasite treatment on tick burdens was not assessed, we assumed that the low level of treatment and its timing late in the year would have had minimal effects on tick burdens on deer [30, 31]. Within this moorland enclosure, eight 0.23 ha plots (four fenced, four unfenced) were set up in 2004/2005 (fence mesh size 20 × 20 cm). The four fenced plots (hereinafter referred to as deer-exclusion plots) excluded deer, while the four unfenced plots (hereinafter referred to as high deer density plots) were accessible to the deer and subjected to high grazing pressure (Additional file 1: Fig. S1). As part of a different study, each plot was divided into five subareas each of approximately 15 m × 15 m to create five habitats: high density birch, low density birch, a single birch in the centre of the plot, high density pine, and a control which was not planted and consisted of *Calluna vulgaris*-dominated heathland. Tree saplings were 9 years old by the time this study started in 2013. To our knowledge, the only hosts permanently present at this site were rodents and birds; other mammals such as hares (*Lepus europaeus*), mustelids, foxes (*Vulpes* vulpes) or badgers (*Meles meles*) were either not present or rare.

### Quantifying *I. ricinus* DON

Questing nymphs were surveyed in 2013, 2014, 2018 and 2019 using a standard dragging method [32] which consists of dragging a 1 m × 1 m square of woollen blanket material over the ground vegetation for 10-m linear transects. In 2013 and 2014, ten transects were surveyed in each of the heather, high density birch and pine habitats for all plots (*n* = 720 transects/year), while in 2018 and 2019, six transects were surveyed in each of the same three habitats (*n* = 432 transects/year). Tick surveys were done in May, July and September between 0900 and 1900 hours. To reduce edge effects, ticks were surveyed at least 2 m away from the fence; ticks tend not to be able to walk horizontally more than this distance [33]. Air temperature and relative humidity were recorded for each transect to be taken account of in statistical models as these factors affect tick activity [34]. Ground vegetation height was recorded at the beginning (at 0 m), middle (at 5 m) and end (at 10 m) of each transect using a sward stick. Questing nymphs were counted, collected and stored at − 20 °C for later pathogen analysis.

### Quantifying rodent activity and tick burdens

To test the effect of vegetation on rodents, and the effect of rodents on NIP (M2—dilution mechanism and M3—ecological cascade mechanism), as well as to quantify tick burdens on rodents (M1—increase in transmission mechanism), rodent activity was estimated in July 2017 and 2018 using a live-trapping method. In 2017, four non-selective Sherman live traps (16 × 5 × 6.5 cm; Sherman, Tallahassee, FL) were baited with oats and placed in each of the five habitats within each plot, at least 2 m away from the edge of the plot, at 10-m spacing, resulting in a total of 20 traps per plot (i.e. 200 trap nights (TN) per deer treatment). Traps were set for three consecutive nights in four of the plots (two fenced/two unfenced) and for two consecutive nights in the remaining four plots. In 2018, four traps were installed for four consecutive nights in the three habitats in which ticks were surveyed (heather, high density birch and pine habitats) in each plot, resulting in 12 traps per plot (i.e. 192 TN per high deer treatment). Traps were activated after 1600 hours and checked every morning before 1000 hours. For all captures, species, sex, weight and approximate age (juveniles or adult) of each individual were recorded. Ticks attached to rodents were counted and collected from around the head and ears and stored in 100% ethanol. All captured rodents were released at the capture site. For analysis, we took the proportion of traps with a capture as our rodent activity index. Both tick burden and rodent captures data were needed to test the dilution effect (M2), which requires a comparison between high deer density plots and deer exclosures in the relative proportion of the larval tick population that feeds on rodents versus deer.

### Measurement of *B. burgdorferi* s.l. prevalence (NIP)

As part of testing all three mechanisms of the effects of high deer density on NIP, and to estimate Lyme disease hazard (DIN), DNA was individually extracted from questing nymphs by using an ammonia extraction method [35]. *Borrelia burgdorferi* s.l. was detected using two methods for this study: nested polymerase chain reaction (PCR) and real-time (quantitative) PCR (qPCR). Ticks collected in 2013 and 2014 were tested using a nested PCR targeting the 5S-23S intergenic spacer region using the protocol described by Rijpkema et al. [36]. Following an issue arising from the nested PCR protocol, samples collected in 2018 and 2019 were tested using a qPCR method. A qPCR protocol on fragments of the * OspA* gene [37] was optimized using the IQ Supermix (Bio-Rad Laboratories, Hercules, USA) in a Stratagene Mx3005P thermal cycler (Agilent, Santa Clara, USA). Each reaction contained IQ Supermix, two primers at 200 nM (B-OspA_modF, AATATTTATTGGGAATAGGTCTAA; B-OspA_borAS, -CTTTGTCTTTTTCTTTRCTTACAAG), the probe (B-OspA_mod-probe, -FAM-AAGCAAAATGTTAGCAGCCTTGA-BHQ-1) at 100 nM and 3 µL of DNA. One positive and one negative control were added for every plate.

We confirmed the correspondence between the two PCR protocols by using 61 known positive samples (by nested PCR, mix of genospecies) and 344 known negative samples. In all cases, results from the qPCR matched those of the nested PCR. Positive samples from the qPCR protocol were subjected to the nested PCR protocol to identify *B. burgdorferi* s.l. genospecies. All PCR products from positive samples were Sanger sequenced to identify the genospecies of *B. burgdorferi* s.l. For analysis, we used the proportion of questing nymphs infected (NIP).

### Statistical analyses

All statistical analyses were performed in R version 3.5.1 (R Core Team, 2013). For generalized linear mixed-effects models (GLMMs) and generalized linear models (GLMs), we tested for potential collinearity between explanatory variables by calculating variance inflation factors (VIFs), and variables for which the VIF was above 4 were discarded from the model [38]. Model selection was done using the dredge function from the MuMIn package [39] based on the corrected Akaike information criterion (AICc) [40]. When appropriate, we conducted post hoc Tukey tests to assess pairwise comparisons between levels of categorical variables.

#### The effects of high deer density on NIP with *B. burgdorferi* s.l.

Our three mechanisms are all concerned with a potential effect of high deer density on NIP. To test for this effect of high deer density, we used a binomial GLMM with a logit link and NIP for *B. burgdorferi* s.l. (three prevalence estimates per habitat and per year) as the response variable. The full model included deer treatment (deer exclusion or high deer density) as our main predictor, as well as month (May, July or September), habitat (high density pine, high density birch or heather control) and year (2013, 2014, 2018 or 2019). An observation-level random effect was included to account for overdispersion [41].

#### Mechanism 1: increase in transmission potential

We investigated whether high deer density led to increased DON, which we hypothesised to be a consequence of high deer density resulting in higher questing larval and nymph density and higher tick burdens on rodents. We used a hurdle GLMM with a Poisson distribution. We tested for zero-inflation using the DHARMa package [42] and we chose a hurdle model as the number of zeros were underestimated with a Poisson GLMM [43]. The response variable was the number of questing nymphs collected per 10 m transect and the full model included deer treatment, month, year, habitat, ground vegetation height, relative humidity, temperature and whether the ground was dry during collection. We also included the interactions between deer treatment and month and between deer treatment and habitat, as the effects of deer on ticks might vary between months and habitat type. Plot number and an observation-level random effect were included, the latter to account for overdispersion [44, 45].

Although we initially planned to compare tick burdens on rodents between deer density treatments, only two rodents were caught in high deer density plots (see M3) so this analysis could not be performed.

#### Mechanisms 2: dilution effect

The dilution effect mechanism implies that the relative proportion of the larval tick population feeding on deer vs rodents (i.e. tick burden × relative abundance of each host type) is higher in high deer density plots compared to exclosures. Testing this formally was not possible because only two rodents were captured in high deer density plots (as predicted by M3), which precluded the fitting of a model of the effects of high deer density on tick burden on rodents. We did not count tick burdens on deer. To evaluate this mechanism, we therefore report descriptive statistics for tick burdens on rodents and relative host abundance.

#### Mechanism 3: ecological cascade

To test the first part of the cascade, that high deer density negatively affects ground vegetation height (M3), we used a GLMM with a Gaussian distribution and ground vegetation height as our response variable. The full model included deer treatment, month and year as fixed effects and a random effect of plot number.

To test the second part of the cascade, that shorter vegetation negatively affects rodent activity, we conducted two analyses, both using a binomial GLMM with a logit-link and our rodent index as the response variable. For the first, the full model included vegetation height, habitat and year as fixed covariates and a random effect of plot number. Vegetation height was averaged per habitat type and per year and we used vegetation height from the heather habitat for the single birch and sparse birch habitats because there was no difference between these three [17]. For the second analysis, testing for a direct impact of deer on rodent activity, the full model included deer treatment, habitat and year as fixed covariates and a random effect of plot number.

To test the last part of the cascade, that rodent abundance positively affects NIP, we used a binomial GLMM. The response variable was NIP for *B. afzelii* and the full model included the number of rodents captured the previous year (data for 2017 and 2018), habitat, year and month. We included an observation-level random effect to account for overdispersion [41].

#### The effects of high deer density on Lyme disease hazard

To test how high deer density affects Lyme disease hazard (DIN), we used a hurdle GLMM with a Poisson distribution, as the number of zeros was underestimated with a Poisson GLMM [43]. The response variable was the DIN and, as this variable was averaged at the plot level (three estimates per habitat and per year), we used an offset for the area surveyed. The full model included deer treatment, month, year, habitat, temperature and whether the ground was dry during tick survey as fixed variables and the plot number and an observation-level random effect were included as random terms.

## Results

Over 4 years of data collection, 12,310 questing nymphs were sampled (Table 1). A random subset of 1000 nymphs was examined under the microscope for species identification using specific keys [46] and all were found to be *Ixodes ricinus.* Thus, we assumed that all questing ticks collected in this study were *I. ricinus.* We tested all 585 questing nymphs collected from deer-exclusion plots and a subset of 1042 from high deer density plots for *B. burgdorferi* s.l. Prevalence (NIP) was 0.9% (95% CI: 0.4–1.6) in high deer density plots and 2.7% (95% CI: 1.6–4.4) in deer-exclusion plots (Table 1). The dominant *B. burgdorferi* s.l. genospecies was the rodent-associated *B. afzelii* (92%, *n* = 23/25) followed by the bird-associated *B. valaisiana* (4%, *n* = 1/25) and *B. garinii* (4%, *n* = 1/23). Over 2 years of trapping, 24 individual bank voles were caught in deer-exclusion plots (6.69/100 TN, SD = 2.41), while only two were captured in high deer density plots (0.51/100 TN, SD = 0.97) and no other rodent species were detected (Table 1).

**Table 1:** Summary of the density of questing nymphs (*DON*), number of bank voles caught per 100 trap nights (*TN*), prevalence of *Borrelia burgdorferi* sensu lato (s.l.) in questing nymphs (*NIP*) and density of nymphs infected with *B. burgdorferi* s.l. (*DIN*) across years and treatments

Treatment	Year^a^	DON (nymphs 10 m^−2^ ± SD)	Bank voles/100 TN ± SD^b^	NIP (%) (95% CI)	DIN (nymphs 1000 m^−2^ ± SD)
High deer density	2013	9.78 ± 9.99	NA	0.56 (0–3.1)	4.81 ± 14.42
	2014	9.67 ± 9.27	NA	0	0
	2017	NA	0.50 ± 1.12	NA	NA
	2018	25.57 ± 25.84	0.52 ± 0.90	0.24 (0–1.3)	3.21 ± 9.64
	2019	6.21 ± 7.60	NA	2.47 (0.9–4.4)	6.12 ± 9.74
	**Average**	**12.10 ± 15.46**	**0.51 ± 0.97**	**0.86 (0.4–1.6)**	**3.86 ± 10.17**
Deer exclusion	2013	0.37 ± 0.84	NA	1.92 (0.2–6.8)	0.96 ± 1.50
	2014	0.54 ± 1.01	NA	4.23 (1.9–7.9)	2.09 ± 3.20
	2017	NA	6.95 ± 2.51	NA	NA
	2018	0.67 ± 1.35	6.25 ± 2.70	0.72 (0–3.9)	0.46 ± 1.39
	2019	0.65 ± 1.11	NA	3.10 (0.9–7.7)	1.96 ± 3.20
	**Average**	**0.53 ± 1.06**	**6.69 ± 2.41**	**2.74 (1.6–4.4)**	**1.41 ± 2.54**

*CI* Confidence interval,* NA *Non-applicable

^a^Ticks were not collected in 2017

^b^Rodent trapping was conducted in 2017 and 2018 only

### The effects of high deer density on NIP with *B. burgdorferi* s.l.

**Fig. 2:**
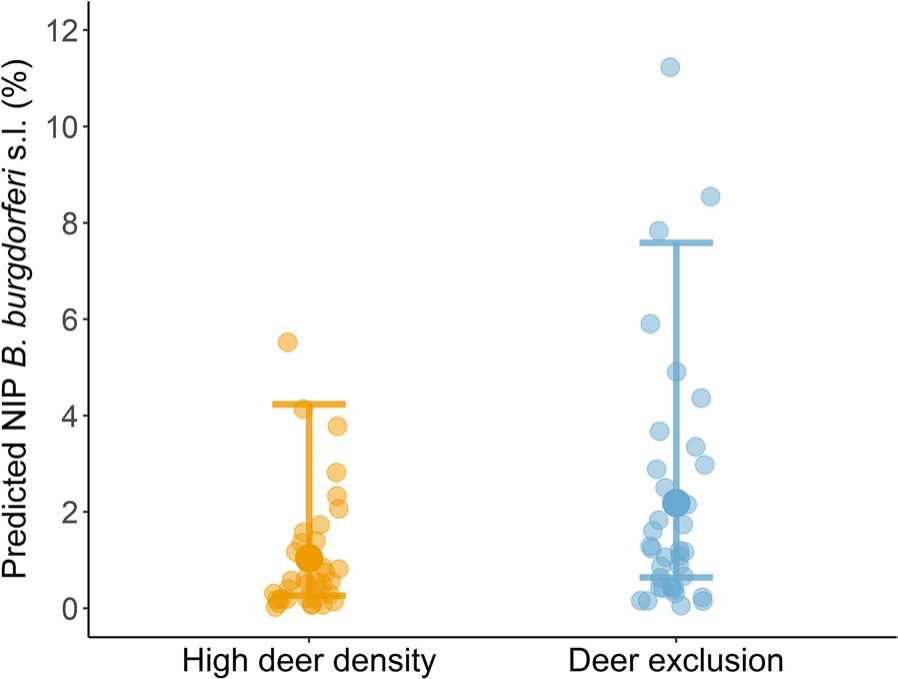
Mechanism 2—dilution effect. NIP for *Borrelia burgdorferi* s.l. (%) [± 95% confidence interval (CI)] in high deer density and deer-exclusion plots

While the best-fit model for NIP only included habitat type, year and month, the second-best model (0.44 increase of AICc compared to the best model) included deer treatment, habitat type and year. Based on this second model, predicted NIP had a lower mean but was statistically indistinguishable in high deer density plots (1.0%, 95% CI: 0.3–4.2) compared to deer-exclusion plots (2.2%, 95% CI: 0.6–7.6) (Fig. 2). NIP also varied across years, habitats and months. The full model results are provided in the Additional file 2: Table S4.

### Mechanism 1: increase in transmission potential

#### Effects of high deer density on DON

**Fig. 3:**
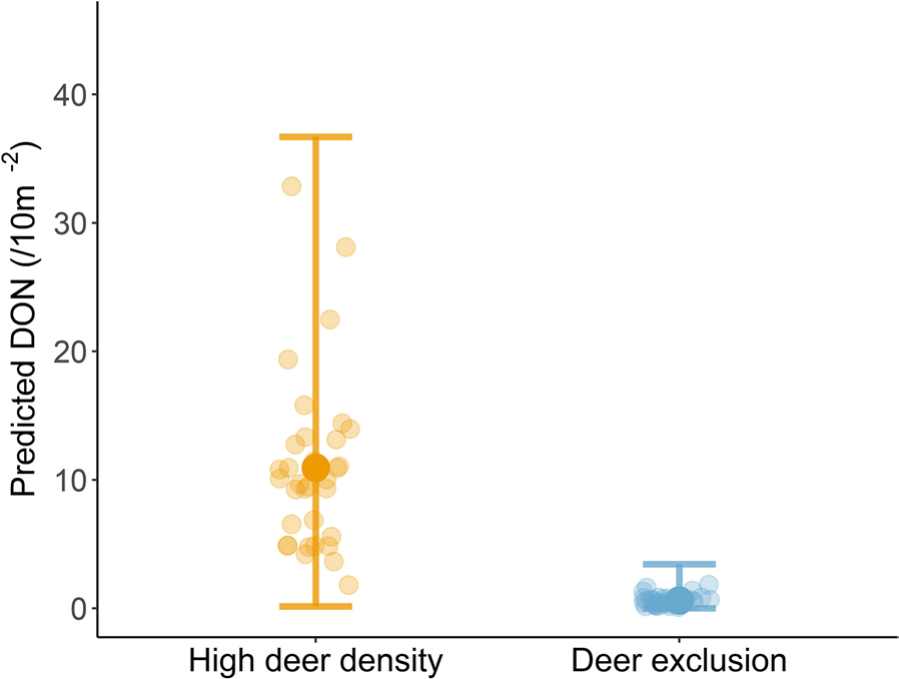
Mechanism 1—increase in transmission potential. DON (nymphs 10 m^−2^) (± 95% CI) in high deer density and deer-exclusion plots. For abbreviations, see Fig. 2

The final model included deer treatment, month, habitat type, year, relative humidity, temperature and whether the ground was wet. On average, predicted DON was 18 times higher in high deer density plots (DON = 10.9, 95% CI: 0.1–36.6) compared to deer-exclusion plots (DON = 0.6, 95% CI: 0–3.4) (Fig. 3). DON varied across months, years and habitats and was influenced by temperature, relative humidity and whether the ground was wet (Additional file 2: Table S1).

#### Effects of high deer density on tick burdens on rodents

Bank voles in deer-exclusion plots (*n* = 24) harboured, on average, 14.2 larvae (range 0–40 larvae per individual) while bank voles in high deer density plots (*n* = 2) had, on average, 17.5 larvae (range 10–25 larvae per individual). Low captures in high deer density plots prevented us from statistically comparing tick burdens between treatments further. No nymphs were found attached to a bank vole.

### Mechanism 2: dilution effect

#### Comparison between high deer density plots and exclosures on the relative proportion of the larval population feeding on rodents vs deer

Based on the number of larvae feeding on the individual bank voles that we caught in deer-exclusion plots (24 individuals feeding 14.2 larvae each, on average), we estimated that these bank voles fed a total of 340.8 larvae. In high deer density plots, the caught bank voles fed 35 larvae (two individuals feeding 17.5 larvae each, on average). These data suggest that 9.7 times more larvae fed on bank voles in deer-exclusion plots compared to high deer density plots. In deer-exclusion plots, no larvae fed on deer (as there were no deer in exclosures). High deer density plots had a deer density of 35.5 deer km^−2^, but we were not able to measure tick burdens on these deer.

### Mechanism 3: ecological cascade

#### Effects of high deer density on ground vegetation

**Fig. 4:**
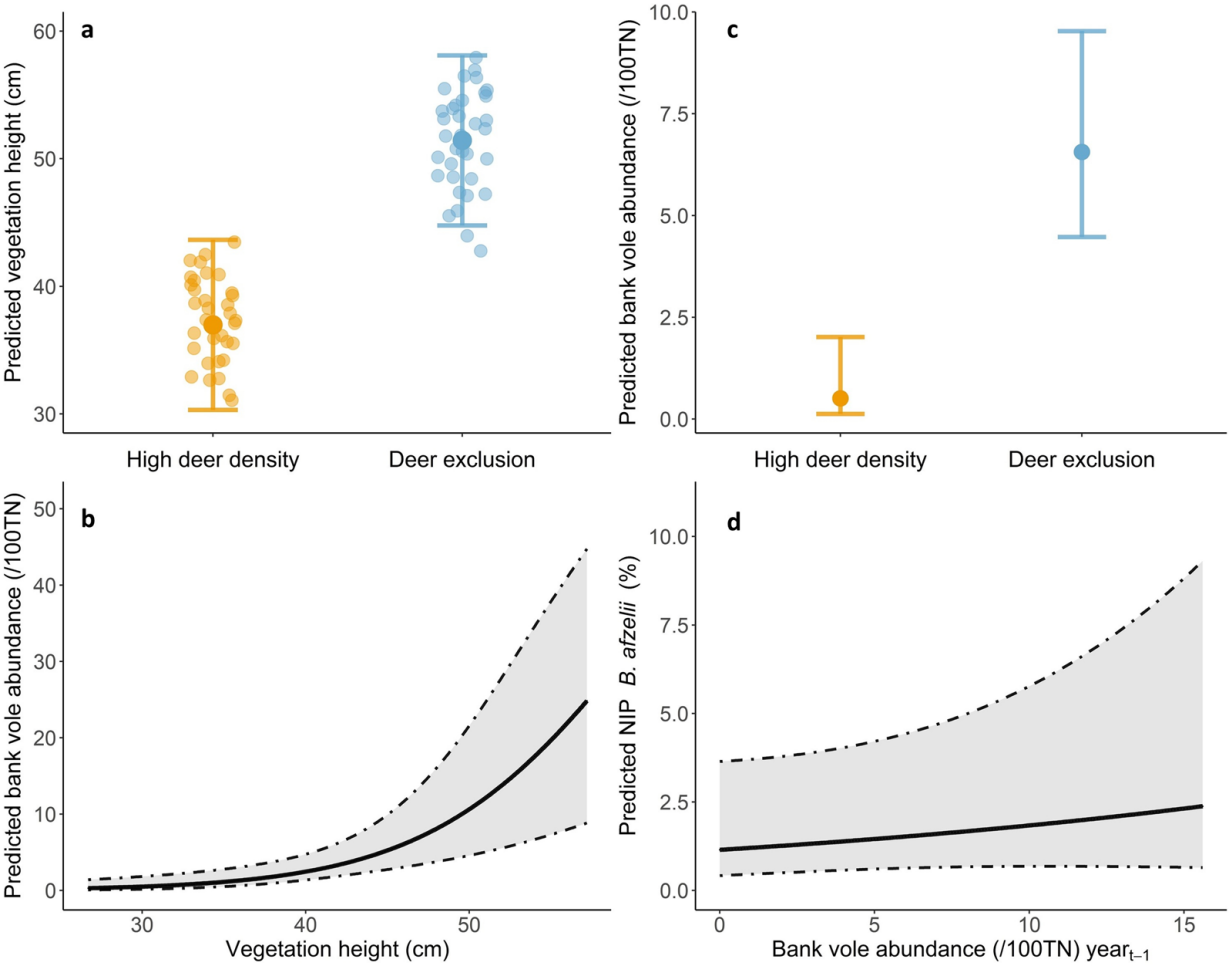
**a**–**d** Mechanism 3—ecological cascades linking high deer density with Lyme disease pathogen prevalence in *Ixodes ricinus* ticks. Graphs show predicted outputs from generalized linear mixed-effects models of ground vegetation height (**a**), bank voles per 100 trap nights (TN) with ground vegetation height (**b**), bank voles per 100 TN in high deer density and deer-exclusion plots (**c**), and NIP with *Borrelia afzelii* (%) with bank vole abundance the previous year (**d**). Error bars and shaded areas represents 95% CI. For other abbreviations, see Fig. 2

The final model included deer treatment, month, habitat type and year. Ground vegetation was predicted to be 14.4 cm shorter in high deer density plots (36.9 cm, 95% CI: 30.3–43.6) compared to deer-exclusion plots (51.4 cm, 95% CI: 44.8–58.1) (Fig. 4a). Ground vegetation height was also influenced by month and differed among habitats (Additional file 2: Table S2).

#### Effects of vegetation height on rodents

The final model included deer treatment and year and the predicted number of bank voles captured was positively correlated with ground vegetation height and increased by 1.17 captures per 100 TN for every 1 cm increase in vegetation height (Fig. 4b). Bank vole capture rate also varied between years (Additional file 2: Table S3).

#### Effects of high deer density on rodent use of space

The final model included deer treatment only and the model predicted that 13 times more bank voles would be captured in deer-exclusion plots (6.6/100 TN, 95% CI: 4.5–9.5) compared to high deer density plots (0.5/100 TN, 95% CI: 0.1–2.0) (Fig. 4c).

#### Effects of rodent capture rate on NIP for *B. afzelii*

While the best-fit model included only year and month, rodents were positively associated with NIP in the second-best model (ΔAICc = 1.9) with an increase in NIP of 0.05% for every vole caught per 100 TN (Fig. 4d) (Additional file 2: Table S4).

### The effects of high deer density on Lyme disease hazard (DIN)

**Fig. 5:**
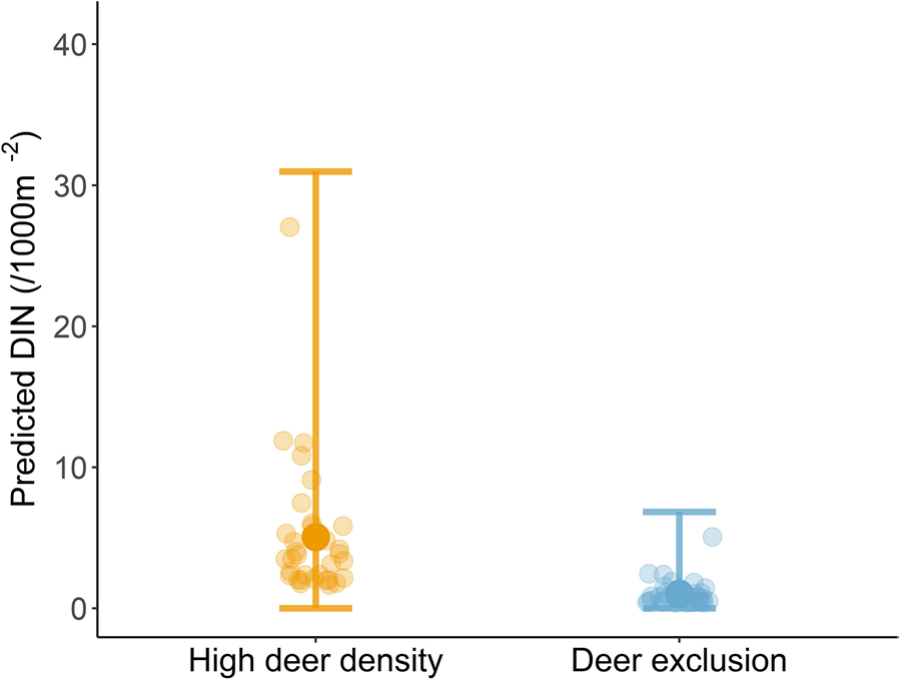
Effects of deer density on DIN (nymphs 1000 m^−2^) (± 95% CI) in high deer density and deer-exclusion plots. For abbreviations, see Fig. 2

The final model included deer treatment, habitat type and temperature. Predicted Lyme disease hazard (DIN) was five times higher in high deer density plots (5.0/1000 m^−2^, 95% CI: 0–30.4) compared to deer-exclusion plots (1.0/1000 m^−2^, 95% CI: 0–7.0) (Fig. 5). DIN was also influenced by habitat and temperature (Additional file 2: Table S5), and the same results were obtained when examining DIN for *B. afzelii* alone (Additional file 3: Table S6).

## Discussion

A fencing experiment allowed us to test three mechanisms (increased transmission potential, dilution and ecological cascades) for how high deer density may affect *B. burgdorferi* s.l. infection prevalence in questing ticks (NIP). Furthermore, we could test how these effects on NIP interact with the role of deer in supporting tick populations and shaping Lyme disease hazard. We found that NIP tended to be lower in high deer density plots along with evidence pointing to a combination of dilution and ecological cascade effects. Despite indications of a lower NIP, Lyme disease hazard (DIN) was five times higher in high deer density plots compared to deer-exclusion plots, due to a strong, positive association between deer and nymph density.

The transmission potential mechanism (M1) predicted that a high deer density would result in an increased tick density, causing higher tick burdens (and therefore increased transmission potential) on individual rodents. We did find a strong positive effect of high deer density on DON, highlighting the importance of deer in driving tick populations, as found in previous studies [16–20].

Several studies have reported positive correlations between DON and tick burdens on rodents [13, 47] and other hosts [48]. This may also have been the case for rodents in our experiment but, as only two bank voles were captured in high deer density plots (as predicted by the ecological cascade mechanism), we were not able to test for differences between deer treatments in individual rodent tick burdens. The dramatic reduction in rodent captures in the high deer density plots is a key finding and also indicates that testing the transmission potential mechanism (M1), which predicts tick burdens for a given transmission host density, could be problematic under these conditions as it is likely that the number of individuals caught would be too low to test this hypothesis.

Our observation that *B. burgdorferi* s.l. prevalence tended to be lower in high deer density plots, despite the estimates being associated with considerable uncertainty, is consistent with predictions of both the dilution effect (M2) and ecological cascade (M3) mechanisms. The dilution effect predicts lower NIP at high deer densities due to a lower proportion of the larval tick population feeding on rodents, which are transmission hosts for *B. burgdorferi* s.l., than on deer that do not transmit the pathogens [20, 22, 24]. This is challenging to test because, ideally, it requires density estimates and tick burdens of the key hosts. We could not obtain tick burden measurements on deer and we caught only two voles in high deer density plots, precluding statistical tests. However, based on differences in vole abundance between deer treatments, we estimated that voles fed almost ten times more larvae in deer-exclusion plots compared to high deer density plots. This compares to no larvae feeding on deer in exclusion plots (as deer were absent), in contrast to the situation in high deer density plots where it is highly likely that most larvae fed on deer. We argue this based on (i) a very high deer density (35.5 deer km^−2^), and (ii) high densities of questing nymphs (implying high numbers of larvae feeding successfully) in high deer density plots. We therefore suggest that a dilution effect was one of the mechanisms operating on NIP in response to high deer densities, as has been previously suggested for *B. burgdorferi* s.l. [20, 22, 24] and predicted for other tick-borne pathogens, including tick-borne encephalitis virus [15] and louping ill virus [49].

While we did not survey other known hosts of larval ticks such as birds or shrews (*Sorex* spp.) [50, 51], previous studies suggest that the effect of high deer density on these groups is also likely to be negative [7, 23, 52]. It is therefore likely that the populations of alternative hosts in general were low in high deer density plots and that the majority of larvae must have been feeding on red deer. In addition, the fact that almost all nymphs that tested positive (92%) were infected with the rodent-associated pathogen *B. afzelii* suggests that other hosts (e.g. birds) contributed little to *B. burgdorferi* s.l. transmission in our system. However, we did not assess whether hedgehogs (*Erinaceus europaeus*) were present, which, as known reservoirs for *B. burgdorferi* s.l. that can transmit *B. afzelii*, could be involved in the transmission cycle [53–55].

To the best of our knowledge, this is the first test of the ecological cascade mechanism of *B. burgdorferi* s.l. prevalence that predicts that grazing pressure from high deer densities could reduce the height of vegetation, which would result in fewer rodents and therefore could lower NIP. We found support for most of the expected ecological links: high deer density resulted in shorter vegetation, which highlighted the effects of deer grazing on vegetation structure [6, 7]. Shorter ground vegetation was associated with fewer bank voles, whereas denser and taller ground vegetation is thought to provide better food, shelter and protection from predators [7, 56]. We captured 13 times fewer bank voles in high deer density plots, consistent with our predictions and with previous work that showed higher densities of bank voles [6] and wood mice [6, 57] in plots that excluded deer. It is possible that direct disturbance from deer (which we did not quantify) could also be a contributing factor to the low density of bank voles in addition to the effect of reduced vegetation cover [7]. However, we could not confirm the last link for the ecological cascade mechanism (i.e. a strong link between rodent activity and NIP) since, although bank vole abundance the previous year was a positive predictor of NIP for *B. afzelii* (Fig. 4d), the effect was statistically weak. The weak association between vole abundance and NIP in this experiment could be due to several factors, including low prevalence of *B. burgdorferi* s.l. overall, providing an insufficient signal for the detection of unequivocal statistical effects. Another contributory factor might have been the small spatial scale of our experiment plots (0.23 ha), facilitating likely movements of rodents between fenced and unfenced plots, which were 30–100 m apart. Quantifying rodent movement through tagging and extended trapping seasons could also aid data interpretation in future studies. These experiments should be replicated on a larger spatial scale to prevent these possible movements and help to untangle the different mechanisms. It would also be interesting to conduct a similar study in a locality with higher density and diversity of rodents to see whether the results can be replicated. Indeed, the overall low abundance and diversity of rodents in our study create specific conditions that might be different from those of other ecosystems.

The small spatial scale of our plots, while a potential issue for confirming a link between rodent abundance and NIP, proved sufficient to demonstrate effects of high deer density on DON, vegetation height and rodent activity, and ultimately, Lyme disease hazard. While a previous meta-analysis of deer-exclusion effects on tick abundance [14] suggested that deer-exclusion areas of at least 2.5 ha may be necessary to have an effect, our results show smaller plots sizes to be sufficient for revealing strong spatial gradients and for testing impacts of deer on ticks, consistent with the findings of other recent studies (Gilbert et al*.* [17] plots of 0.2–0.25 ha; Mysterud et al*.* [18] plots of 0.04 ha).

Irrespective of the mechanisms driving NIP, the most critical parameter governing public health and policy importance for Lyme disease is the DIN (= NIP × DON), which is the key proxy for Lyme disease hazard in the environment [29]. DIN was five times higher in high deer density plots compared to exclosures due to deer having a strong positive effect on DON. Similarly, other studies have found a positive correlation between deer density and Lyme disease hazard [24, 25] or Lyme disease incidence in humans [18]. Using an experimental system with high deer densities (35.5 deer km^−2^), we were able to demonstrate that the role of deer as tick reproduction hosts is, for high deer densities, more important than their role in lowering NIP through dilution and ecological cascade mechanisms in shaping Lyme disease hazard.

In contrast to our experiment, previous studies have shown Lyme disease hazard to be associated with higher densities of transmission hosts [25, 58]. However, such an association requires enough vectors in the environment to transmit the pathogen effectively [59], whereas in our experimental situation, the plots with high densities of transmission hosts did not have deer, and therefore had few ticks to aid transmission. Thus, we might expect Lyme disease hazard to be highest in an environment supporting both high numbers of transmission hosts and tick reproduction hosts. However, based on our findings and previous research showing that high deer densities have strong negative effects on rodent abundance [7, 8], such a combination may not commonly occur in nature.

Our results highlight the need for a systems approach to studying such a complex disease system, where different host types might affect each other’s densities through habitat modification, or by other means such as predation [3]. Gaps in knowledge that can now be addressed include testing for cascading effects of deer on *B. burgdorferi* s.l. prevalence at landscape scales, with the inclusion of a full range of intermediate deer densities, to investigate non-linearities and thresholds of effects of deer and to test whether the mechanisms supported here operate in a non-experimental setting.

## Conclusions

In conclusion, we found evidence suggesting that high deer density could lower Lyme disease pathogen prevalence by a combination of dilution and ecological cascade effects. Despite this, Lyme disease hazard was five times higher in high deer density plots due to a strong positive effect of deer on tick density. This study is, to our knowledge, the first to test for cascading effects of deer on *B. burgdorferi* s.l. prevalence via grazing and suppression of rodent abundance. This study highlights the need for a systems approach to understand disease dynamics and risks that could arise from complex ecological interactions between host types and habitat.

## Supplementary Information

**Additional file 1.** Experimental design. **Figure S1.** This additional file presents a picture of the experimental design with the different plots.

**Additional file 2.** Summary tables for GLMs and GLMMs. **Table S1.** Summary table from the selected model to explain what causes variations in questing nymph abundance. GLMM focussing on questing nymph abundance. **Table S2.** Summary table from the selected model to explain what causes variations in ground vegetation height, ground vegetation density and tree height. **Table S3.** Summary table from the selected model to explain what causes variations in bank vole abundance. **Table S4.** Summary table from the selected model to explain what causes variations in nymphal infection prevalence. **Table S5.** Summary table from the selected model to explain what causes variations in the density of infected nymphs.

**Additional file 3.** GLMM for the density of nymphs infected with *Borrelia afzelii*. **Table S6.** Summary table from the selected model to explain what causes variations in the density of nymphs infected with *B. afzelii.*

## Data Availability

The datasets used and/or analysed during the current study are available from the corresponding author on reasonable request.
