# Supplementary material for: Experimental evidence for opposing effects of high deer density on tick-borne pathogen prevalence and hazard
Source: Parasit Vectors. 2021 Sep 30;14:509. doi: 10.1186/s13071-021-05000-0 (PMC8485466; doi:10.1186/s13071-021-05000-0)

Experimental evidence for opposing effects of high deer density on tick-borne disease pathogen prevalence and hazard

Sara Gandy[1,2*], Elizabeth Kilbride[1], Roman Biek[1], Caroline Millins[1,3*], Lucy Gilbert[1]

[1]Institute of Biodiversity, Animal Health and Comparative Medicine, University of Glasgow, Glasgow, UK

[2]The James Hutton Institute, Craigiebuckler, Aberdeen, UK

[3]Institute of Infection, Veterinary and Ecological Sciences, University of Liverpool, Liverpool, UK.

*Correspondence: sara38.gandy@gmail.com

Additional file 1: Figure S1. Experimental design

Description: Experimental design for this study representing the four fenced plots (Plots 1, 4, 6 and 7) and high deer density plots (Plots 2, 3, 5 and 8). Coloured rectangles represent the different habitats within each plot. Not to scale.

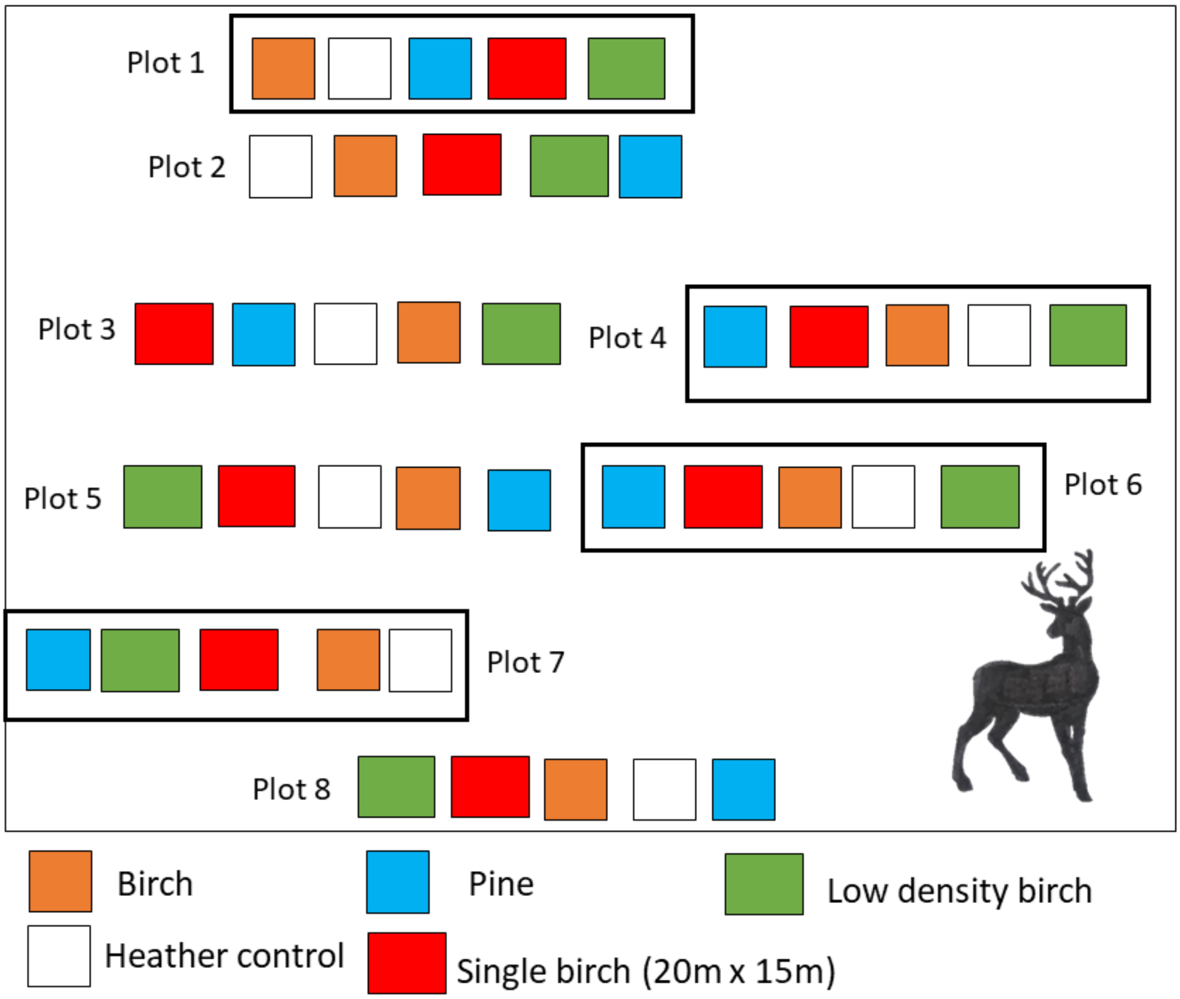

Supplement: Supplementary file 1 — Additional file 1. Experimental design. Figure S1. This additional file presents a picture of the experimental design with the different plots. [file 13071_2021_5000_MOESM1_ESM.pdf]
