# Supplementary material for: Experimental evidence for opposing effects of high deer density on tick-borne pathogen prevalence and hazard
Source: Parasit Vectors. 2021 Sep 30;14:509. doi: 10.1186/s13071-021-05000-0 (PMC8485466; doi:10.1186/s13071-021-05000-0)
Supplement: Supplementary file 2 — Additional file 2. Summary tables for GLMs and GLMMs. Table S1. Summary table from the selected model to explain what causes variations in questing nymph abundance. GLMM focussing on questing nymph abundance. Table S2. Summary table from the selected model to explain what causes variations in ground vegetation height, ground vegetation density and tree height. Table S3. Summary table from the selected model to explain what causes variations in bank vole abundance. Table S4. Summary table from the selected model to explain what causes variations in nymphal infection prevalence. Table S5. Summary table from the selected model to explain what causes variations in the density of infected nymphs. [file 13071_2021_5000_MOESM2_ESM.pdf]

Experimental evidence for opposing effects of high deer density on tick-borne disease pathogen prevalence and hazard

Sara Gandy[1,2*], Elizabeth Kilbride[1], Roman Biek[1], Caroline Millins[1,3*], Lucy Gilbert[1]

[1]Institute of Biodiversity, Animal Health and Comparative Medicine, University of Glasgow, Glasgow, UK

[2]The James Hutton Institute, Craigiebuckler, Aberdeen, UK

[3]Institute of Infection, Veterinary and Ecological Sciences, University of Liverpool, Liverpool, UK.

*Correspondence: sara38.gandy@gmail.com

Additional file 2: Summary tables for GLMs and GLMMs

Description: Full summary tables from the selected models to explain what causes variations in questing nymph abundance (Table S1), vegetation height (Table S2), vegetation density (Table S2), tree height (Table S2) bank vole abundance (Table S3) nymphal infection prevalence (Table S4) and the density of nymphs infected with *B. burgdorferi* s.l. (Table S5).

Table S1 Summary table from the selected model to explain what causes variations in questing nymph abundance

	Estimate (Log)	Std Error	z-value	p-value
**Conditional model**				
Intercept	2.46	0.10	24.93	<0.001
Deer treatment: deer exclusion	-2.35	0.13	-18.69	<0.001
Month				
May	-0.24	0.08	-2.79	0.005
September	-0.29	0.07	-4.35	<0.001
Year				
2014	0.04	0.06	0.69	0.49
2018	0.89	0.08	11.08	<0.001
2019	-0.23	0.10	-2.41	0.02
Habitat				
Heather	-0.83	0.06	-13.27	<0.001
Pine	-0.16	0.06	-2.77	0.006
Relative humidity	-0.18	0.03	-5.28	<0.001
Ground wet: yes	-0.49	0.18	-2.77	0.006
Temperature	-0.10	0.04	-2.36	0.02
**Zero-inflation model**				
Intercept	0.04	0.06	0.69	<0.001
Deer treatment: deer exclusion	0.89	0.08	11.08	<0.001
Month: May	-0.23	0.10	-2.41	<0.001
Month: September	-0.83	0.06	-13.27	0.99
Habitat: heather	-0.16	0.06	-2.77	<0.001
Habitat: pine	-0.18	0.03	-5.28	0.97
Year: 2014	-0.49	0.18	-2.77	0.01
Year: 2018	-0.10	0.04	-2.36	<0.001
Year: 2019	-0.57	0.20	-2.81	<0.001
Ground wet: yes	1.86	0.33	5.70	<0.001

Table S2 Summary table from the selected model to explain what causes variations in ground vegetation height, ground vegetation density and tree height

	Estimate (Log)	Std Error	t-value
**Vegetation height**			
Intercept	32.90	3.39	9.69
Deer treatment: deer exclusion	14.46	4.76	3.04
Month			
May	-4.59	0.43	-10.65
September	-1.45	0.43	-3.38
Year			
2014	4.46	0.44	10.10
2018	7.81	0.51	15.32
2019	6.82	0.52	13.15
Habitat			
Heather	2.75	0.43	6.41
Pine	1.19	0.43	2.77

Table S3 Summary table from the selected model to explain what causes variations in bank vole abundance

	Estimate (Log)	Std Error	z-value	p-value
**Model with deer treatment**				
Intercept	-5.27	0.71	-7.44	<0.001
Deer treatment: deer exclusion	2.62	0.74	3.54	<0.001
**Model with vegetation height**				
Intercept	-9.51	1.86	-5.11	<0.001
Vegetation height	0.16	0.05	3.57	<0.001
Year: 2018	-2.32	0.78	-3.00	0.003

Table S4 Summary table from the selected model to explain what causes variations in nymphal infection prevalence

	Estimate (log)	Std Error	z-value	p-value
**Model focussing on deer treatment (*B. burgdorferi* s.l.) H2 – dilution hypothesis**				
Intercept	-6.20	0.96	-6.47	<0.001
Deer treatment: deer exclusion	0.77	0.52	1.48	0.14
Month				
May	-1.07	0.83	-1.30	0.20
September	0.90	0.50	1.79	0.07
Habitat				
Heather	1.05	0.70	1.49	0.14
Pine	1.45	0.62	2.34	0.02
Year				
2014	0.72	0.74	0.97	0.33
2018	-1.03	0.95	-1.09	0.28
2019	1.02	0.70	1.46	0.14
**Model focussing on bank vole abundance (*B. afzelii*) H3 – ecological cascades hypothesis**				
Intercept	-7.41	1.22	-6.08	<0.001
Bank vole abundance $year_{t-1}$	0.05	0.05	1.06	0.29
Month				
May	0.90	1.23	0.73	0.47
September	2.48	1.06	2.34	0.02
Year: 2019	1.90	0.79	2.41	0.02

Table S5 Summary table from the selected model to explain what causes variations in the density of nymphs infected with *B. burgdorferi* s.l.

	Estimate (Log)	Std Error	z-value	p-value
**Conditional model**				
Intercept	-5.85	0.75	-7.84	<0.001
Deer treatment: deer exclusion	-1.76	0.28	-6.26	<0.001
Habitat				
Heather	0.05	0.42	0.12	0.91
Pine	0.83	0.39	2.12	0.03
Temperature	0.10	0.05	2.27	0.02
**Zero-inflation model**				
Intercept	-1.61	1.10	-1.47	0.14
Ground wet: no	3.10	1.15	2.71	0.007
