# Supplementary material for: Experimental evidence for opposing effects of high deer density on tick-borne pathogen prevalence and hazard
Source: Parasit Vectors. 2021 Sep 30;14:509. doi: 10.1186/s13071-021-05000-0 (PMC8485466; doi:10.1186/s13071-021-05000-0)
Supplement: Supplementary file 3 — Additional file 3. GLMM for the density of nymphs infected with Borrelia afzelii. Table S6. Summary table from the selected model to explain what causes variations in the density of nymphs infected with B. afzelii. [file 13071_2021_5000_MOESM3_ESM.pdf]

Experimental evidence for opposing effects of high deer density on tick-borne disease pathogen prevalence and hazard

Sara Gandy[1,2*], Elizabeth Kilbride[1], Roman Biek[1], Caroline Millins[1,3*], Lucy Gilbert[1]

[1]Institute of Biodiversity, Animal Health and Comparative Medicine, University of Glasgow, Glasgow, UK

[2]The James Hutton Institute, Craigiebuckler, Aberdeen, UK

[3]Institute of Infection, Veterinary and Ecological Sciences, University of Liverpool, Liverpool, UK.

*Correspondence: sara38.gandy@gmail.com

Additional file 3: GLMM for the density of infected nymphs with *B. afzelii*

The mixed effect model used to investigate the effect of fencing, habitat, month and year on Lyme disease hazard for *B. burgdorferi* s.l. was conducted for *B. afzelii* infections only. As seen from Table 7, results are similar to the other model and Lyme disease hazard is predicted to be higher in high deer density plots (predicted DIN=1.18, 95%CI: 0-6.24) compared to deer exclusion plots (predicted DIN=2.26,95%CI: 0-18.50).

Table S6 Results from the selected model to explain what causes variations in the density of infected nymphs for B. *afzelii*. ΔAICc indicates the increase in AICc after dropping each variable from the model

	Estimate (Log)	Std Error	z-value	p-value	ΔAICc
**Conditional part**					
Intercept	-4.49	0.36	-17.48	<0.001	
Deer treatment: deer exclusion	-1.46	0.19	-7.64	<0.001	18.6
Habitat					9.2
Heather	-0.20	0.32	-0.60	0.55	
Pine	0.89	0.27	3.23	0.001	
**Zero inflation part**					
Intercept	-1.14	1.14	-1.00	0.32	
Deer treatment: deer exclusion	-1.16	0.73	-1.60	0.11	
Ground wet: Yes	3.44	1.21	2.86	0.004	
